# Bioresponsive Hydrogel for On-Demand Nonhormonal Contraception

**DOI:** 10.3390/gels11110858

**Published:** 2025-10-27

**Authors:** Giovanni M. Pauletti, Pankaj Dwivedi, Ping Li, Aluet Borrego-Alvarez, Hidemi S. Yamamoto, Julie Lewis, Sarah Alobaidi, Amel Ibrahim, Raina N. Fichorova, Celia M. Santi

**Affiliations:** 1 Department of Pharmaceutical and Administrative Sciences, St. Louis College of Pharmacy, University of Health Sciences and Pharmacy in St. Louis, St. Louis, MO 63110, USA; 2 Department of Obstetrics & Gynecology, Washington University School of Medicine, St. Louis, MO 63110, USA; 3 Department of Obstetrics, Gynecology and Reproductive Biology, Harvard Medical School, Boston, MA 02115, USA; 4 School of Pharmacy and Pharmaceutical Sciences, Trinity College Dublin, The University of Dublin, D04 V1W8 Dublin, Ireland

**Keywords:** physical contraceptive barrier, drug-free, female-initiated, partner-independent

## Abstract

The utility of bioresponsive multifunctional hydrogel compositions for biomedical applications is rapidly increasing due to the diverse array of biological stimuli that can profoundly alter physicochemical gel properties that benefit therapeutic interventions. The purpose of this research is to explore a bioresponsive hydrogel as a drug-free bioengineering concept to fortify the natural physical contraceptive barriers at the cervicovaginal junction. The results of this research demonstrate that a hydrogel comprising 4% (*w*/*w*) Carbopol^®^ 974P and 4% (*w*/*w*) polyvinylpyrrolidone (CP4%/PVP4%) undergoes bioresponsive structural changes in the presence of simulated seminal fluid, pH 7.7, (SFS) that increases the work required to spread the gel under physiologically relevant vaginal conditions. Combination of this bioresponsive hydrogel with liquified human semen at a volumetric ratio of 1:5 dramatically reduces in vitro sperm migration by 97%. Simultaneously, total sperm motility decreases from 72.0 ± 9.9% to 7.9 ± 13.7%, which is significantly below the WHO criteria defined for male fertility. Safety assessments performed in vitro and in vivo underline a robust vaginal safety profile comparable to approved vaginal products. Moreover, the results from an exploratory animal study performed with female New Zealand White rabbits suggest that the drug-free physical barrier established intravaginally after exposure of the bioresponsive CP4%/PVP4% hydrogel to alkaline semen seems at least equivalent in the prevention of pregnancy in vivo to the VCF^®^ Gel (Apothecus Pharmaceuticals, Ronkonkoma, NY, USA), a marketed spermicidal on-demand product containing nonoxynol-9.

## 1. Introduction

Global health statistics reveal that nearly half of all pregnancies occur in advance of a preferred timeframe or are completely unplanned. This is predicted to correlate with increased child mortality, decreased maternal health, and limited gender equality, particularly in low- and middle-income countries. Even in industrialized countries such as the United States, it is estimated that approximately 30% of reproductive age women who do not desire pregnancy often find themselves without viable options that meet their needs [1,2,3]. As current contraceptive methods do not appear to deliver the desired impact and/or gender norms limit a woman’s ability to access/use existing preventive tools, the challenge of the future is to develop innovative, female-initiated contraceptive technologies. To facilitate adoption by end users, these new solutions must be affordable, socially acceptable, and provide desired protection from unintended pregnancy without exposing women to the health risks associated with systemic distribution of contraceptive hormones.

For most women, the need for pregnancy prevention changes during their reproductive life cycle. Therefore, no single product will appeal to everyone. Instead, women want choices to protect themselves throughout different stages of their lives [4]. Among the coitally-dependent (i.e., on-demand), nonhormonal contraceptive methods, the male condom is most frequently used. However, evidence from behavior research underlines that partner opinion strongly correlates with the likelihood of contraceptive use or nonuse [5,6]. Among female-initiated, nonhormonal contraceptive on-demand methods, barrier devices such as the female condom and diaphragm reduce the risk of unintended pregnancy most effectively. Low user acceptability and high cost have constrained wide-spread use of these devices [7]. Alternatively, women can select a locally acting spermicide such as nonoxynol-9 (N-9) or the acidic buffer gel composition Phexxi^®^ (Evofem Biosciences, San Diego, CA, USA) as a hormone-free vaginal product to prevent unwanted pregnancy. The reported failure rate of up to 25% renders these marketed on-demand products among the least effective of all modern family planning methods, only slightly more reliable than “traditional” methods such as withdrawal or fertility tracking [8]. Moreover, N-9 gels are now suspected of altering the vaginal microflora and inducing immunoinflammatory reactions at the cervicovaginal mucosa that increase the risk of acute and recurring vaginal infections as well as sexually transmissible infections such as HIV/AIDS [9].

Physiologically, the increased viscoelastic properties of the human cervical mucus during the luteal phase of the menstrual cycle establish an effective natural barrier to pregnancy by physically restricting sperm movement from the vaginal cavity towards the fallopian tube, a required step for successful oocyte fertilization [10]. In addition to this natural physical contraceptive barrier, the acidic vaginal milieu represents an inhospitable environment for sperm that limits capacitation and viability, thereby reducing fertility [11,12]. The overall objective of this project is to develop bioresponsive multifunctional hydrogel formulations suitable for establishing a highly viscous physical barrier at the cervicovaginal junction and, simultaneously, maintaining an acidic vaginal environment in the presence of alkaline semen to fortify the natural contraceptive barriers without the need for inclusion of a pharmacologically active drug molecule. To accomplish the desired contraceptive endpoint, this study describes the selection of pharmaceutically acceptable formulation excipients, assessment of bioresponsive viscoelastic properties and gel phase pH values under simulated physiological conditions in vitro, estimation of cervicovaginal safety and contraceptive efficacy in vitro, as well as safety and contraceptive efficacy in vivo using the rabbit model.

## 2. Results and Discussion

### 2.1. Bioresponsive Physicochemical Gel Properties

**Figure 1:**
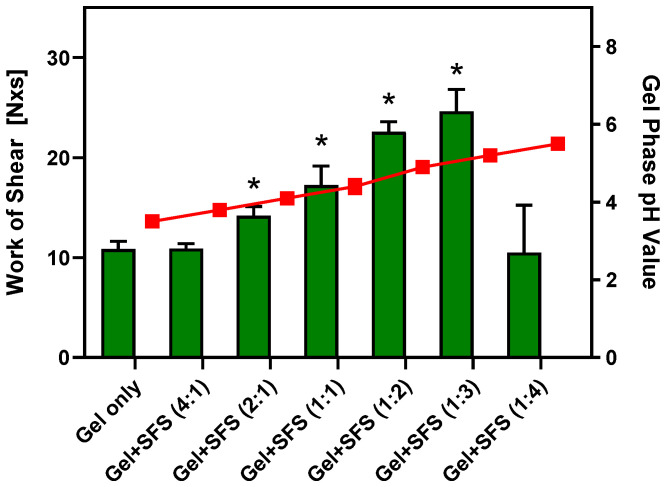
Physicochemical properties of a 4% (*w*/*w*) Carbopol^®^ 974P NF/4% (*w*/*w*) polyvinylpyrrolidone (CP4%/PVP4%) hydrogel in the presence of seminal fluid simulant, pH 7.7 (SFS). Work of shear (green bars) and gel phase pH value (red symbols) were measured after dilution of the hydrogel with SFS at volumetric gel/SFS ratios ranging from 4:1 to 1:4. Data are shown as mean ± SD (n = 6). * = significantly different from undiluted gel (*p* < 0.03, one-way ANOVA and Dunnett’s multiple comparisons test).

To identify a lead composition for the fabrication of hydrogels that exhibit the desired bioresponsive viscoelastic properties, exploratory preformulation studies were performed using various FDA-approved acidic polymers such as carboxymethylcellulose, polycarbophil, alginic acid, and carbomers in combination with selected viscosity-enhancing polymers. Guided by these results, the combination of Carbopol^®^ 974P NF (CP) and polyvinylpyrrolidone (PVP) at a gel concentration of 4% (*w*/*w*) each was selected for this study. Figure 1 summarizes the results of in vitro physicochemical performance assessments of this hydrogel composition after dilution with seminal fluid simulant, pH 7.7 (SFS) that was prepared according to Owen & Katz [13].

The design selected for these experiments was based on important physiological parameters relevant to the intended vaginal administration, including the average intravaginal pressure exerted by soft tissue surrounding the vaginal pelvic floor in a supine position [14] and human semen properties such as volume and pH value [15]. Moreover, as intravaginal contraceptive gel formulations are generally administered at a fixed dose (i.e., 3–5 mL), but human semen volume is highly variable [15], the spreadability and gel pH values of the hydrogel formulation were measured in the presence of SFS using volumetric gel/SFS ratios ranging from 4:1 to 1:4. Comparative assessment of the spreadability data revealed that the CP4%/PVP4% hydrogel underwent significant changes in structure and viscoelastic properties after a 2-fold dilution with SFS as demonstrated by the greater work of shear recorded. The resistance of the hydrogel to spread under the applied force continues to increase upon exposure to SFS until a peak value of ~25 N × s is reached at a volumetric gel/SFS ratio = 1:3. It is acknowledged that these spreadability assessments were performed at room temperature due to experimental limitations. Therefore, the quantitative changes in the work of shear recorded after dilution with SFS may not adequately represent the physiological intravaginal conditions as a consequence of temperature-dependent viscoelastic properties of the CP4%/PVP4% hydrogel. Using a correlation established experimentally for a series of viscosity standards and the work of shear measured under equivalent in vitro conditions as described for the hydrogel formulation (Figure S1), the results from these in vitro experiments suggest that the viscoelastic properties of the CP4%/PVP4% hydrogel, when combined with alkaline seminal fluid at a volumetric ratio greater than 2:1, exceed the rheological properties of the cervical mucus measured in healthy, non-pregnant human subjects at the end of the progesterone-dominated luteal phase of the menstrual cycle, which is considered to provide natural contraception [10]. In parallel, the CP4%/PVP4% hydrogel composition demonstrated a robust acidic buffer capacity after volumetric dilution with alkaline SFS. The pH value of the gel phase moderately increased from 3.5 ± 0.1 to 5.2 ± 0.1 after 3-fold dilution with SFS at which point it is reported that sperm motility decreases below WHO fertility standards within 15 min [12].

Consistent with the pH-dependent rheological behavior of carbomers [16], it is hypothesized that the presence of alkaline SFS increases ionization of the carboxylic acid groups within the acrylic acid polymer backbone and, thus, reduces intramolecular hydrogen bonding. Consequently, polymer strands begin to uncoil and extend under the influence of electrostatic repulsion forces, which can increase the volume of microgel particles up to 1000-fold [17]. Combination of the polyacrylic acid-based CP with high-molecular weight PVP also provides an opportunity to form interpolymer complexes. Lau and Mi described such complexes stabilized by strong hydrogen bonding in thin film mixtures of polyacrylic acid and PVP using Fourier transform infrared spectroscopy (FTIR) and high-resolution ^13^C solid-state nuclear magnetic resonance spectroscopy [18]. In the presence of water, formation of interpolymer complexes between polyacrylic acid and PVP is critically dependent on the solution pH value to ensure limited dissociation of the polyacid that serves as the proton-donating substrate. Conversely, aggregation, complexation, and complex disintegration between polyacrylic acid and PVP are highly sensitive to polymer concentration and molecular weight, respectively [19,20]. Since the work of shear required to spread the CP4%/PVP4% hydrogel after a 4-fold dilution with SFS substantially decreased, it is conceivable that unfavorable pH conditions and/or polymer concentrations resulted in the dissociation of these interpolymer complexes. Alternatively, the presence of a high cation concentration such as Na^+^ ions in SFS may have significantly reduced the electrostatic repulsion of negatively charged carboxylate groups within CP, thereby limiting pH-dependent thickening of the microgel [21,22]. Future studies combining static and dynamic light scattering with high-resolution analytical techniques such as FTIR are expected to provide more insight into the mechanisms of pH-dependent CP ionization and hydrogen bonding between CP and PVP that drive this bioresponsive change in rheological properties.

**Figure 2:**
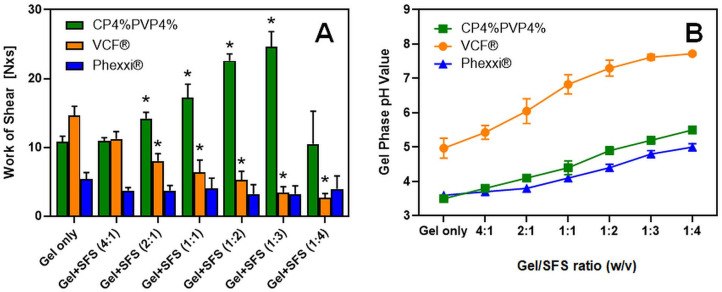
Comparative physicochemical properties of vaginal contraceptive gel compositions after exposure to seminal fluid simulant, pH 7.7 (SFS). (**A**) shows the work of shear quantified after dilution of the CP4%/PVP4% hydrogel, the marketed spermicidal VCF™ Gel containing nonoxynol-9, or the acid buffering gel Phexxi^®^ with SFS at volumetric gel/SFS ratios ranging from 4:1 to 1:4. (**B**) summarized the corresponding gel phase pH values measured for each gel composition after dilution with SFS. Data are shown as mean ± SD (n = 6). * = significantly different from undiluted gel (*p* < 0.03, one-way ANOVA and Dunnett’s multiple comparisons test.

In contrast to the CP4%/PVP4% hydrogel, the results for the marketed contraceptive VCF^®^ and Phexxi^®^ gel formulations shown in Figure 2 reveal dramatically different spreadability and gel pH profiles under similar experimental conditions. The greatest work of shear required to spread the VCF^®^ Gel was recorded in the absence of SFS.

Exposure to increasing volume fractions of alkaline SFS as a semen surrogate appears to continuously decrease the viscoelastic properties of the VCF^®^ Gel, arriving at ~20% of the work of shear measured for the gel only when diluted with SFS to a 1:4 gel/SFS ratio (i.e., 2.7 ± 0.6 N × s vs. 14.6 ± 1.4 N × s). Among the list of inactive ingredients disclosed for this contraceptive formulation containing 4% (*w*/*v*) of the spermicide N-9, carboxymethylcellulose and PVP are the only gel-forming polymers included. Kristmundsdóttir and colleagues studied the effect of different buffer compositions on the viscosity of carboxy-methylcellulose/PVP hydrogel compositions [23]. The results from this study confirmed a decrease in gel viscosity upon interactions with lactate, citrate/lactate, or maleate buffers. The authors concluded that the presence of buffer ions may shield the charged carboxyl groups of the acidic polymer component and, thus, lead to a less expanded polymer network that negatively affects rheological gel properties. This hypothesis seems consistent with our explanation for the reduced work of shear measured for the CP4%/PVP4% hydrogel at a 1:4 gel/SFS ratio (see Figure 1). However, it appears that polymer concentrations in the marketed VCF^®^ Gel were optimized to decrease gel viscosity upon exposure to alkaline semen with the intent to rapidly release N-9 from the hydrogel, as this chemical spermicide solely provides contraceptive efficacy by damaging the sperm’s cellular membrane [24].

Combination of the acidic buffer gel Phexxi^®^ with increasing SFS volume fractions, on the other hand, did not seem to affect the rheological gel properties within the gel/SFS ratios studied, as the work of shear required to spread Phexxi^®^ in the absence of SFS was not significantly different from the corresponding value measured at the Phexxi^®^/SFS ratio = 1:4 (i.e., 5.4 ± 0.9 N × s vs. 4.0 ± 1.9 N × s). The presence of alginic acid and xanthan gum in the marketed Phexxi^®^ composition suggests the mucoadhesive and viscosity-enhancing functions of these polymers. The viscosity of alginic acid gels typically depends on ionotropic gelation, a process driven by divalent cations or high alkalinity [25]. Since these factors are absent in Phexxi^®^, the gel viscosity must instead be governed by its mucoadhesive properties, which are essential for retaining the acidic buffer composition in the vaginal cavity. Therefore, strong mucoadhesion is the critical performance characteristic in Phexxi^®^ that is achieved by including these polymers in the hydrogel. This conclusion is further supported by comparing the gel pH profiles for the various hydrogels after dilution with SFS (see Figure 2B). The gel pH value of Phexxi^®^ measured in the presence of a 4-fold volumetric SFS ratio was most acidic at pH 5.0 ± 0.1, followed by the bioresponsive CP4%/PVP% hydrogel at pH 5.6 ± 0.1, and the VCF^®^ Gel at pH 7.7 ± 0.1. Combined, the results from these physicochemical gel assessments that were performed under physiologically relevant in vitro conditions, seem to fully align with the predicted principle of operation underlying the contraceptive efficacy of each hydrogel composition: CP4%/PVP4% hydrogel = bioresponsive physical barrier that impedes sperm migration from the vagina to the uterus, VCF^®^ Gel = chemical barrier that kills sperm cells in the vaginal cavity mediated by the surface-active spermicide N-9, and Phexxi^®^ = chemical barrier that kills sperm cells in the vaginal cavity due to a buffered acidic gel environment.

### 2.2. Vaginal Safety In Vitro

**Figure 3:**
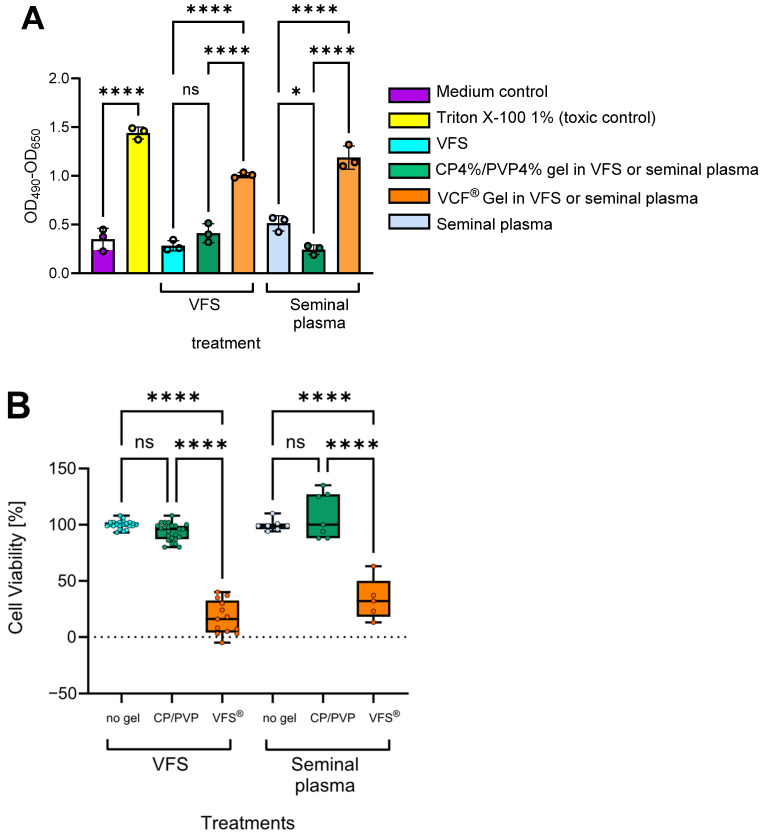
Effects of various topical treatments on epithelial cell viability and membrane integrity in the EpiVaginal™ tissue colonized with *L. crispatus*. Release of lactate dehydrogenase (LDH) was assessed by enzyme activity (photometry assay described in the Method section) in culture supernatant after 24 h incubation at 37 °C. (**A**) summarizes the optical density (OD) of enzyme activity released from the basolateral surface of triplicate tissues (cumulative biological replicates = 24) representing one of eight experiments with two different batches of the CP4%/PVP4% gel and VCF^®^ Gel blended with vaginal fluid simulant (VFS) or human seminal plasma, with medium serving as baseline (low control) and Triton X-100 1% serving as 100% toxicity control (high control). Data are reported as mean ± SD. (**B**) depicts box plots demonstrating vaginal cell viability based on the LDH results from all 8 experiments (cumulative biological replicates n = 114) calculated as 100% − 100 × (test − mean of low control/mean of high control − mean of low control). Raw data are available on FigShare. * *p* < 0.05, **** *p* < 0.0001 from two-way ANOVA and Tukey’s multiple comparisons test.

**Figure 4:**
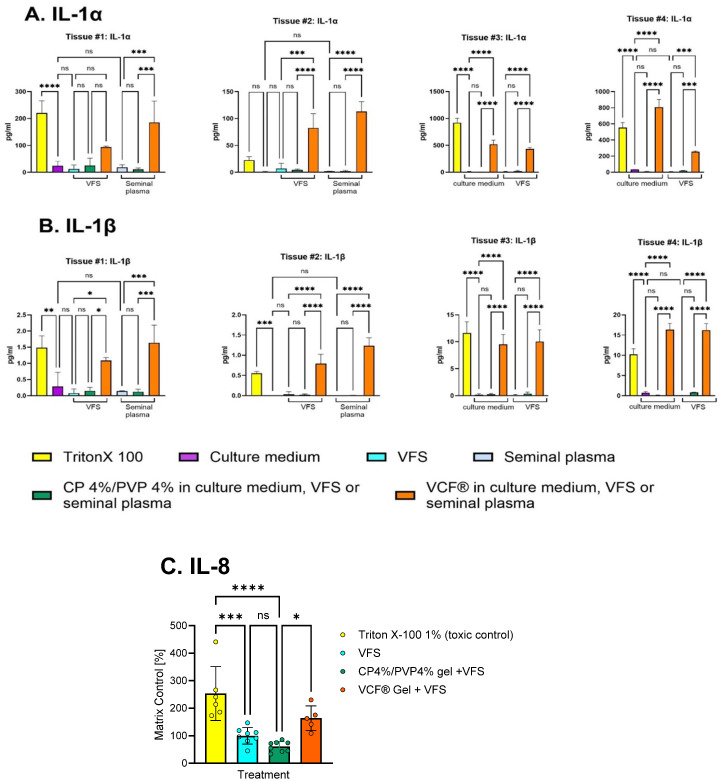
Effect of various topical treatments on release of the proinflammatory cytokines IL-1α (**A**), IL-1β (**B**), and IL-8 (**C**) by the EpiVaginal™ tissue colonized with *L. crispatus*. Cytokines were measured after a 24 h incubation with the following test articles: Trion X-100 1% (high control), med = cell culture medium (low controls), CP4%/PVP4% hydrogel, and VCF^®^ Gel in the presence of medium, vaginal fluid simulant (VFS), or seminal plasma (SP). Bars represent mean ± SD from biological triplicates or duplicates in experiments with four different bioengineered tissue batches. * *p* < 0.05, ** *p* < 0.01, *** *p* < 0.001, **** *p* < 0.0001, ANOVA and Tukey’s multiple comparisons test.

To assess the vaginal safety of the bioresponsive CP4%/PVP% hydrogel in vitro, epithelial tissue viability was assessed after a 24 h exposure using a Lactobacillus-colonized EpiVaginal™ VEC-100 model [26]. The results from comparative experiments measuring release of the intracellular enzyme lactate dehydrogenase (LDH) demonstrated that the CP4%/PVP4% hydrogel is nontoxic in the presence of both vaginal fluid simulant and seminal plasma matrix (Figure 3A). In contrast, the marketed contraceptive VCF^®^ Gel significantly reduced the viability of this co-cultured in vitro vaginal tissue model (*p* < 0.0001). Analysis of IL-1α and IL-1β concentrations in cell culture fluids collected from the basolateral compartment of the same Lactobacillus-colonized vaginal tissue model revealed similarly preserved cell integrity and lack of proinflammatory activity in the presence of the CP4%/PVP4% hydrogel when compared to medium/matrix controls (Figure 4A,B). In contrast, the levels of proinflammatory cytokines IL-1α and IL-1β were significantly increased after treatment with Triton X-100 (toxic control) and the VCF^®^ Gel.

Similarly, the CP4%/PVP4% hydrogel appeared non-inflammatory based on significant downregulation of the proinflammatory chemokine IL-8 (Figure 4C). Only basolateral concentrations were assessed, because apical release of biomarker proteins into the gel phase is not a reliable measure of tissue physiology due to (i) the variable degree of physicochemical assay interferences with products applied apically, and (ii) potential product-independent tissue vulnerability that occurs when attempting to collect small volumes of apically applied gels and media [27,28]. The MTT proliferation test was not chosen for toxicity assessment because it is considered unreliable and not reproducible for assessing toxicity of full-strength formulations (such as gels and high-viscosity derivatives) due to established physicochemical interferences. The LDH cytotoxicity assay was chosen because it has been validated for gel formulations [27,28]. A 24 h period is typically used for in vitro assessment of causative effects on cytokine production to allow the time needed for de novo protein synthesis in response to irritating, toxic chemicals and proinflammatory signal transduction and accumulation of well-detectable protein levels [27,28,29,30]. The maximum 24 h time interval is dictated by the 24 h proliferation cycle of the epithelial cells.

**Figure 5:**
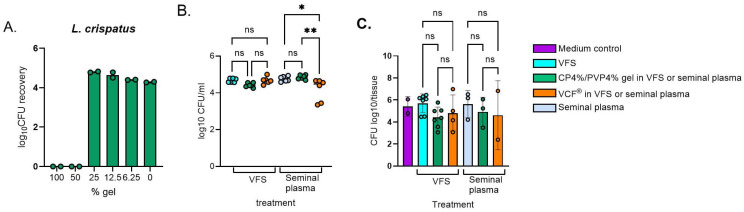
Recovery of vaginal bacteria (*L. crispatus*) after exposure to the CP4%/PVP4% gel. Experiments were performed in the presence of various biological matrices (i.e., vaginal fluid simulant, VSF and human seminal plasma, SP) using undiluted (**A**) or 5-fold diluted gel (**B**) in the absence of epithelial cells. (**C**) summarizes bacterial recovery after a 24 h colonization of the EpiVaginal™ VEC-100 tissue. Bacterial recovery was quantified by colony-forming units (CFU) on agar plates. Data are presented as CFU/tissue from three independent experiments with biological replicates in each experiment. * *p* < 0.05, ** *p* < 0.01, ANOVA and Tukey’s multiple comparisons test.

To explore the effect of gel compositions on bacterial growth assessed by colony-forming units (CFU) on agar plates, a bacterial suspension of *L. crispatus* was first mixed with a 2-fold dilution of the CP4%/PVP4% gel. Bacterial growth was suppressed when mixing with undiluted gel, possibly due to viscosity presenting the uniform spread of bacteria over the agar surface. The bacterial recovery was fully restored at a 4-fold dilution of the gel (Figure 5A). Based on these findings and the fact that the gel will be diluted with biological fluids at the time of bioresponsive contraceptive efficacy, further experiments were conducted with a 5-fold diluted gel formulation (Figure 5B), which demonstrated full bacterial recovery in the presence of vaginal fluid simulant and seminal plasma. Beneficial bacterial survival and colonization was maintained in the presence of the gel when diluted in VFS or seminal plasma (Figure 5C).

**Figure 6:**
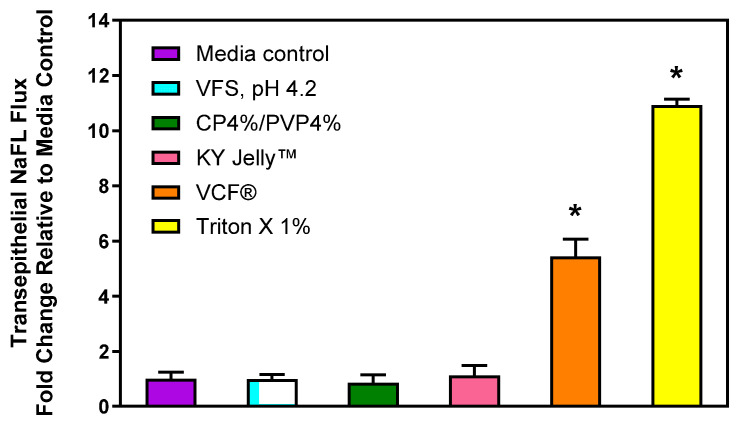
Effect of various topical treatments on the physical barrier properties of the EpiVaginal™ tissue model in vitro. Transepithelial flux of sodium fluorescein (NaFL) was quantified after a 6 h incubation at 37 °C using fluorescence spectroscopy and normalized to media-treated control tissue. Data are reported as mean ± SD (n = 4–6). * = significantly different from media-treated control tissue (*p* < 0.001 one-way ANOVA and Dunnett’s multiple comparisons test).

To explore the impact of these gel compositions on the physical barrier properties of the vaginal mucosa that protect underlying tissues from exposure to microorganisms and xenobiotics, transepithelial flux of sodium fluorescein (NaFL) was quantified. NaFL is a small, hydrophilic solute that permeates the mucosal barrier by passive diffusion, predominantly via the paracellular pathway [31,32]. After a 6 h incubation of the EpiVaginal™ tissue model with serum-free cell culture media, 11.2 ± 4.8% of the NaFL dose administered at t = 0 min to the apical side of the vaginal tissue barrier was recovered after 120 min in the basolateral receiver compartment. In comparison to these control data, paracellular permeability of the EpiVaginal™ tissue model was not significantly altered following incubation with vaginal fluid simulant, pH 4.2 (VFS), the CP4%/PVP4% gel, or KY Jelly™ (Reckitt, Parsippany, NJ, USA), which was selected as a positive safety comparator (Figure 6).

These results suggest that the bioresponsive polymeric gel comprising 4%CP and 4%PVP is unlikely to increase the risk of greater vaginal permeability in vivo. In contrast, transepithelial NaFL flux significantly increased more than 5-fold after a 6 h exposure to the spermicidal VCF^®^ Gel containing 4% (*w*/*v*) N-9. Previously, Ayehunie and co-workers assessed the effects of 0.002–0.2% (*w*/*v*) N-9 on vaginal permeability in vitro and reported a NaFL flux of >20% at the lowest concentration studied [31]. These in vitro data underline the vaginal safety concerns raised regarding topical use of N-9-containing spermicidal vaginal gels, which also have been documented in humans [33].

### 2.3. Contraceptive Efficacy In Vitro

**Figure 7:**
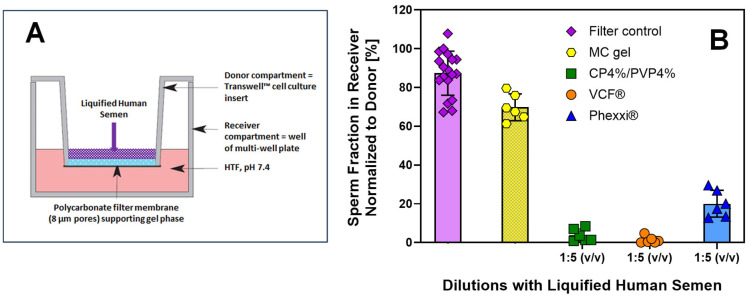
Contraceptive efficacy assessment in vitro using human sperm samples. (**A**) shows the experimental design of in vitro sperm migration studies using Transwell™ cell culture inserts as described in detail in the Methods. (**B**) summarizes the results of in vitro permeation studies performed in the presence and absence of various gel formulations. Individual symbols represent the results of each measurement, whereas the bar summarizes the data as mean ± SD for each condition assessed.

Following demonstration of favorable in vitro bioresponsive physicochemical properties and vaginal safety of the CP4%/PVP4% hydrogel, we next sought to quantify its contraceptive efficacy in vitro. For that purpose, a sperm migration assay was adapted from the literature [34] to quantify the contraceptive efficacy of this drug-free hydrogel composition using semen samples obtained from human volunteers. Due to the opaque optical properties of the CP4%/PVP4% hydrogel, conventional microscopic assessment of sperm counts and motility in the presence of the gel formulation was not feasible. However, the experimental design shown in Figure 7A enabled microscopic comparison of the sperm number and motility before and after interaction with the gel barrier. It is noted that all semen samples obtained from healthy volunteer donors for this study were compliant with the normal threshold values defined for male fertility according to the WHO manual [15], specifically sperm concentration (≥1.5 × 10^7^/mL) and total motility (>40%). However, due to large inter-individual variability in these parameters that included sperm concentrations ranging from 1.7 × 10^7^/mL to 4.1 × 10^8^/mL, corresponding total motility values from 52.6% to 88.7%, and semen pH value from 7.1 to 8.5; thus, it was essential to perform the series of assessments using the same donor sample. Subsequent comparison of semen parameters after interaction with a selected gel barrier that were normalized to the filter control as outlined in the Methods allowed for statistical comparison of the results between replicates obtained from different sperm donors.

In the absence of a gel barrier (i.e., filter control), the sperm recovery for 18 different donor samples in the receiver compartment was 87.4 ± 11.4% when compared to the total number of sperm added to the donor compartment at t = 0 min (Figure 7B). The results from these control experiments suggest that the polycarbonate membrane of the Transwell™ insert exhibiting an 8-µm pore size does not seem to represent a significant barrier for sperm to migrate from the donor to the receiver compartment within 60 min. This is consistent with the expectation for a normal semen sample with average sperm head dimensions of <5 μm in length and 2.5–3.2 μm in width [15]. Furthermore, the total motility of sperm in the receiver compartment after traversing the polycarbonate membrane is not significantly different from the total motility measured for the same semen sample that was added to the donor compartment (67.6 ± 15.9% vs. 72.0 ± 9.9%, n = 18). It was, therefore, concluded that filter-normalized sperm recovery rate and total motility assessments of sperm in the receiver compartment after traversing a gel barrier are suitable quantitative in vitro surrogate markers for comparative assessment of the contraceptive efficacy of the various gel compositions studied.

In addition to the gel compositions with predicted contraceptive efficacy, sperm migration using the dual-chamber Transwell™ assay was also assessed across a 1% (*w*/*w*) methylcellulose (MC) gel barrier, which represents a clinically valuable alternative to mid-cycle cervical mucus for sperm penetration (i.e., Kremer) tests [35]. The results shown in Figure 7B confirm that close to 70% of the sperm cells added to the donor compartment successfully migrated across the MC gel barrier into the receiver compartment within 60 min. As this sperm recovery rate is significantly lower than for the filter control, it underlines that the viscoelastic properties of the MC gel are sufficient to filter a fraction of up to 30% of sperm cells that do not appear to exhibit adequate motility to penetrate a mid-cycle cervical mucus barrier. However, the total motility of sperm that successfully migrated across the MC gel barrier was comparable to that of sperm moving from the donor to the receiver compartment in the absence of a gel barrier (i.e., filter control). These findings are aligned with the clinical utility of in vitro sperm functional tests such as the sperm penetration test for diagnosing male factor infertility as a prognostic factor for fertilization outcome in couples undergoing advanced assisted reproductive technologies [36]. Conversely, the sperm migration results obtained in the presence of the MC gel barrier can serve as a quantitative comparator to gauge the contraceptive efficacy of the CP4%/PVP4% gel, VCF^®^ Gel, and Phexxi^®^, respectively.

Table 1 summarizes the results from in vitro sperm migration assays across gel barriers with predicted contraceptive efficacy. To quantitatively delineate the contribution of the gel compositions on contraceptive efficacy in vitro, all experiments were performed at a gel/liquified semen ratio = 1:5 (*v*/*v*). Comparison of the total motility of the sperm samples that were added to the donor compartment revealed that the differences were not considered to be statistically significant. This outcome provided the statistical foundation to rank the various gel compositions according to the total sperm motility measured in the receiver compartment and to comment on the relative “contraceptive potency” of the hydrogels.

The sperm recovery rates visualized in Figure 7B for the CP4%/PVP4% gel, VCF^®^ Gel, and Phexxi^®^ unquestionably confirm that all three hydrogel formulations dramatically reduce the ability of human sperm to migrate across a gel barrier. Closer evaluation of the corresponding motility data for sperm collected from the receiver compartment allows for a more nuanced interpretation of these experimental results. While sperm migration rate in the presence of the Phexxi^®^ gel barrier was reduced to ~1/3 of the corresponding value measured in the presence of the MC gel barrier, statistical comparison revealed that the CP4%/PVP4% gel and the VCF^®^ Gel were significantly more effective in reducing the number of sperm cells that successfully migrated into the receiver compartment. Both the CP4%/PVP4% gel and the VCF^®^ Gel reduced the sperm number by ~20- or 50-fold when compared to the corresponding number measured in the presence of the MC gel barrier. However, there was no statistically significant difference between the two contraceptive hydrogel compositions. It is noted that the gel pH value measured at a gel/liquified semen ratio = 1:5 (*w*/*v*) was comparable for the CP4%/PVP4% gel to the value measured at the gel/SFS ratio =1:4 (*w*/*v*). For the VCF^®^ Gel and Phexxi^®^, however, the composition of the human semen samples resulted in a more acidic gel pH value for the VCF^®^ Gel and a more alkaline gel pH value for Phexxi^®^, which may have affected the outcome of these in vitro efficacy studies. The total motility data recorded for sperm in the receiver compartment varied for the CP4%/PVP4% gel between 0 and 33.3%, for the VCF^®^ Gel between 0 and 4.7%, and for Phexxi^®^ between 8.4 and 63.4%, respectively. Considering sperm recovery rate and total motility in the receiver compartment, a predictive “contraceptive potency” ranking emerged, favoring VCF^®^ Gel > CP4%/PVP4% gel > Phexxi^®^ using the WHO criteria defined for male fertility [15]. Notably, it is emphasized that the bioresponsive CP4%/PVP4% gel is the only hydrogel composition that accomplishes contraceptive efficacy via a drug-free physical barrier while exhibiting a favorable vaginal safety profile in vitro.

### 2.4. Vaginal Safety In Vivo

To explore the validity of the favorable in vitro performance data obtained for the bioresposive CP4%/PVP4% gel under in vivo conditions, a small animal pilot study was designed using female New Zealand White rabbits. This nonclinical model was selected as it currently represents the best available small laboratory animal species that is accepted for extrapolation of reproductive safety data to humans [37,38]. Since the pH value of the vaginal cavity in nonclinical animal species, including the rabbit, is between pH 6.5–7.5 [39], each doe received a vaginal douche with 5 mL of VFS, pH 4.2, prior to the test article administration, so safety parameters could be assessed under pH conditions relevant to humans. It was confirmed previously that this vaginal douche procedure does not induce any significant changes in the vaginal safety parameters assessed. All animals survived the procedure to scheduled euthanasia on gestation day (GD) 12. Furthermore, there were no clinical observations recorded during the entire study duration of 12 days that were considered treatment related. As outlined in Table 2, dermal scoring of the external genitalia four hours after vaginal dose administration identified one animal in the sham control group and two animals in the CP4%/PVP4% gel treatment group with vulval grade 1 erythema.

It is noted that none of the animals dosed with the N-9-containing VCF^®^ Gel showed any signs of similar vulval erythema despite earlier in vitro finding that suggested modulation of vaginal mucosal barrier properties by this contraceptive gel formulation (see Figure 6). However, since the light redness in sham- and CP4%/PVP4% gel-treated animals was absent at a subsequent observation performed at 24 h, it was concluded that these transient effects were incidental to administration procedures to the delicate vaginal cavity using custom-made applicators. Body weight in all animals increased during the 12-day gestation period, without any significant difference between treatment groups. Gross pathological evaluation of the reproductive organs after scheduled euthanasia on GD12 only revealed pink discoloration of the vaginal canal in one animal of each the CP4%/PVP4% gel and VCF^®^ Gel treatment group. No visible lesions were identified in the vagina, cervix, uterus, or ovary/oviduct. As this observation was limited to one animal only in a given dosing group, it was considered incidental to administration procedures to the delicate vaginal cavity using custom-made applicators. Collectively, these results demonstrate a robust in vivo vaginal safety profile of the CP4%/PVP4% gel using this nonclinical animal model that is indicative of favorable safety in humans.

### 2.5. Contraceptive Efficacy In Vivo

The predicted contraceptive mechanism of the CP4%/PVP4% gel relies on a bioresponsive change in viscoelastic gel properties upon exposure to alkaline semen that creates an impermeable physical barrier for sperm to reach the oocyte and, consequently, prevents fertilization. The results from our in vitro contraceptive efficacy studies using the dual-chamber Transwell™ assay demonstrated the ability of the CP4%/PVP4% gel to significant impede sperm migration and reduce total motility to levels consistent with infertility (see Figure 7). As pregnancy rate is the clinical endpoint required for validation of contraceptive efficacy, the rabbit safety study was designed to also serve as an exploratory efficacy pilot using artificial insemination following vaginal gel administration. Ovarian and uterine examinations after scheduled euthanasia on GD12 were used to determine pregnancy rate in each treatment cohort. The results summarized in Table 3 reveal that 9 out of the 10 sham-treated animals were confirmed pregnant on GD12, with a mean group number of 6.5 embryos. In contrast, vaginal administration of the bioresponsive CP4%/PVP4% gel prevented pregnancy in all animals treated (i.e., 0 pregnancy in 10 CP4%/PVP4–treated animals). When compared to the VCF^®^ Gel, where two pregnancies were confirmed, with a mean group number of 1.7 embryos, the results from this exploratory animal study suggest that the contraceptive efficacy of the CP4%/PVP4% in vivo seems at least equivalent to the efficacy of the marketed spermicidal on-demand product. As the number of corpora lutea determined in each cohort was comparable, it is concluded that ovulation was induced equally in all treatment groups. Therefore, the reduced pregnancy rate in gel-treated animals is attributed to the predicted contraceptive mechanism of each of the gel compositions applied prior to artificial insemination (i.e., physical barrier for the CP4%/PVP4% gel and spermicidal activity of N-9 for the VCF^®^ Gel, respectively).

## 3. Conclusions

Results from this study demonstrate that the CP4%/PVP4% hydrogel undergoes bioresponsive structural changes in the presence of seminal fluid simulant, pH 7.7, that dramatically increase the work required to spread the gel under physiologically relevant vaginal pressure. The combination of the CP4%/PVP4% hydrogel with liquified human semen dramatically reduced in vitro sperm migration and total sperm motility to levels below the WHO criteria defined for male fertility [15]. The vaginal mucosal safety assessment of the CP4%/PVP4% hydrogel performed using the human-derived EpiVaginal™ VEC-100 tissue model demonstrated a lack of toxicity as indicated by low levels of extracellular LDH and IL-1α, which are markers of membrane integrity and viability [29,40]. The absence of increased production and release of IL-1α, IL-β, and IL-8 in the presence of confirmed beneficial bacterial colonization also demonstrated a favorable non-inflammatory safety profile compared to the marketed VCF^®^ Gel. The favorable safety features were equally maintained in the presence of vaginal fluid simulant and human seminal plasma, thus meeting in vitro preclinical milestones of contraceptive product development.

Vaginal safety assessment performed in female New Zealand White rabbits underlines a robust in vivo safety profile without any significant clinical observations. Mild vulval erythema was recorded in some animals that received the CP4%/PVP4% hydrogel. However, the transient nature of these events suggests that they were likely incidental to administration procedures to the delicate vaginal cavity using custom-made vaginal gel applicators. Vaginal administration of the CP4%/PVP4% hydrogel prior to artificial insemination of female New Zealand White rabbits was effective in preventing pregnancy in all animals treated. The results from this exploratory animal study suggest that the drug-free physical barrier established intravaginally after exposure of the bioresponsive CP4%/PVP4% hydrogel to alkaline semen seems at least equivalent in the prevention of pregnancy in vivo to the VCF^®^ Gel, a marketed spermicidal on-demand product containing nonoxynol-9. Future studies involving a larger number of animals are required to statistically compare safety and contraceptive efficacy. To further explore the viability of this bioresponsive hydrogel composition for female-initiated, nonhormonal, on-demand contraception, it will be essential to address critical development questions such as formulation scale-up and stability, as well as understand end user desirability and acceptability.

## 4. Materials and Methods

### 4.1. Materials

Carbopol^®^ 974P NF polymer (CP, MW = 3 × 10^6^ g/mol) was a gift from Lubrizol (Wickliffe, OH, USA). The supplier of this polymer was not involved in the study design, data collection, or interpretation of study results. Polyvinylpyrrolidone (PVP, MW = 1.3 MDa), methylcellulose (4000 cP), bovine serum albumin, sodium fluorescein (NaFL), Triton X-100, Rogosa agar, human tubular fluid (HTF), Hanks’ Balanced Salts, and human chorionic gonadotropin were obtained from MilliporeSigma Corp. (St. Louis, MO, USA). The EpiVaginal™ Tissue Model (VEC-100) was obtained from MatTek Corp. (Ashland, MA, USA). Transwell^®^ permeation chambers comprising a filter insert with a 6.5 mm-in-diameter polyester membrane exhibiting 8-μm pores (#3422) were purchased from Corning Life Sciences (Durham, NC, USA). The legally marketed vaginal lubricant KY Jelly™ (Reckitt, Parsippany, NJ, USA) and the contraceptive VCF^®^ (Apothecus Pharmaceuticals, Ronkonkoma, NY, USA) and Phexxi^®^ (Evofem Biosciences, San Diego, CA, USA) gel products were procured from a local drug store. All other chemicals and solvents were of high purity or analytical grade and used as received.

### 4.2. Methods

#### 4.2.1. Gel Fabrication

A standard batch of bioresponsive CP4%/PVP4% hydrogel was prepared by gradually suspending 2.4 g of CP and 2.4 g of PVP under constant stirring at 600 rpm (Caframo SDC1850 overhead stirrer, Georgian Bluffs, ON, Canada) in 55.2 g of ultrapure water (Milli-Q, EMD Millipore Corp., Burlington, MA, USA) at room temperature for 60 min. The pH value of the hydrogel was measured using the DeltaTrak^®^ Pocket ISFET pH Meter, which is equipped with an ion-sensitive field effect transistor pH sensor and an integrated temperature sensor (DeltaTrak, Inc., Pleasanton, CA, USA). The pH value of the fabricated hydrogel batches was 3.2 ± 0.1. To increase biocompatibility with the normal physiological vaginal environment [41], the gel pH value was adjusted to pH 3.5 using dropwise addition of 5N NaOH solution under continuous stirring. Similarly, a conventional sol–gel fabrication process was used to prepare the 1% (*w*/*w*) methylcellulose gel for sperm migration studies. Fully hydrated gel preparations were used for experiments after an overnight gelation time at room temperature.

#### 4.2.2. Gel Performance In Vitro

##### Spreadability

To quantify changes in structural and viscoelastic gel properties under various simulated intravaginal conditions, spreadability of the fully hydrated CP4%/PVP4% gel was quantified at room temperature using the TA–XTPlus texture analyzer (Stable Microsystems, Godalming, UK) fitted with the cone-cap assembly. For a single measurement, 0.5 g of the hydrogel was filled into the 45° acrylic cap and combined with seminal fluid simulant (SFS), pH 7.7, which was prepared according to Owen & Katz [13], at volumetric gel/SFS ratios ranging from 4:1 to 1:4 (*w*/*v*). After a 60 s incubation at room temperature, the coaxially aligned 45° cone was lowered at a test speed of 1 mm/s (i.e., compression mode) until a maximum load force of 5 N was reached. This value was selected as it represents the average physiological intravaginal pressure exerted by the soft tissue surrounding the vaginal pelvic floor in a supine position [14]. The area under the force-distance curve is equivalent to the total work performed to spread (or shear) the gel formulation. Consequently, the work of shear is a suitable quantitative in vitro parameter for comparing the dynamics of gel spreading under various simulated vaginal conditions. The pH value of the gel phase at the end of each spreadability experiment was measured using the DeltaTrak^®^ Pocket ISFET pH Meter. Each experiment was performed using a fresh gel sample.

##### Vaginal Mucosal Safety

To assess whether topical administration of the CP4%/PVP4% hydrogel induces undesired vaginal safety concerns, cytotoxicity, changes in vaginal barrier properties, release of proinflammatory cytokines, and compatibility with Lactobacillus crispatus—a major component of the beneficial human vaginal microbiome [42,43]—were quantified using the human-derived EpiVaginal™ VEC-100 tissue model as previously described and widely accepted for preclinical vaginal safety assessment [27,44]. Each test article was applied to the apical surface of the vaginal tissue model undiluted as supplied. The release of safety biomarkers of tissue toxicity and immune function was monitored in the basolateral compartment of the 3D tissue model by sampling in a 24 h interval for signs of toxicity, including release of the intracellular enzyme lactate dehydrogenase (LDH) and selected proinflammatory cytokines. Treatment with 1% (*v*/*v*) Triton X-100 served as a positive toxicity control (high control) and medium alone served to measure levels of spontaneous LDH release from the tissues (low control) over the exposure interval. Percent viability was assessed as described in Equation (1):

100% − 100 × (test − mean of low control)/(mean of high control − mean of low control) (1)

Cytokine assays were performed on the basolateral supernatants from the EpiVaginal™ tissue representing a 24 h period of cytokine release. The assay kits were obtained from Meso Scale Discovery (MSD, Gaithersburg, MD, USA) and completed using the MSD imager 600, following procedures under accreditation by the College of American Pathologists. The less abundant proteins IL-1α, IL-1β were assessed simultaneously in undiluted culture media using an MSD U-Plex. IL-8, which is more abundantly expressed at steady state by the VEC-100 tissues as compared to the other markers, was assessed at 10-, 50-, or 500-fold dilutions as needed to fit within the detection range of the MSD V-plex assay. CFU counts of bacteria upon mixing with test articles or epithelia-associated CFU counts were assessed as previously described after lysing each tissue and seeding the lysates on Rogosa agar [26].

Changes in the barrier function of the vaginal epithelium were estimated by quantifying transepithelial flux of sodium fluorescein (NaFL) using a protocol adapted from Koumangoye and colleagues [45]. Briefly, EpiVaginal™ tissue samples were incubated for 6 h with test formulations at 37 °C and washed three times using 300 μL of prewarmed VFS, pH 4.2, before 300 μL of a 0.01% (*w*/*v*) NaFL solution prepared in VFS, pH 4.2, was added to the apical compartment of each tissue sample. The basolateral receiver compartment was filled with 500 μL of Hanks’ Balanced Salt solution, pH 7.4. After a 60 min incubation at 37 °C, the fluorescence intensity of an aliquot removed from the basolateral receiver compartment was measured using the BioTek Synergy HTX Multimode Microplate Reader (Agilent, Santa Clara, CA, USA) with excitation and emission wavelength set at 480 nm and 530 nm, respectively. All experiments were performed in quadruplicate and results normalized to media-treated EpiVaginal™ tissue samples. For comparison, similar experiments were performed with the vaginal lubricant KY Jelly™ and the contraceptive VCF^®^ Gel that contains 4% (*w*/*w*) of nonoxynol-9. A 1% (*v*/*v*) Triton X-100 solution prepared in VFS, pH 4.2, was used as positive control.

##### Sperm Migration

As an in vitro surrogate of contraceptive efficacy, human sperm migration properties in the presence and absence of the CP4%/PVP4% gel barrier were quantified using the Transwell^®^ dual-compartment system. In an adaptation of a protocol published earlier by Chen and co-workers [34], 20 µL of the CP4%/PVP4% hydrogel were deposited onto the filter membrane of the donor compartment that was preincubated overnight in VFS, pH 4.2, at 37 °C. The gel was allowed to spread evenly for 60 min at 37 °C before the receiver compartment was filled with 600 µL of non-capacitating human tubal fluid (HTF) comprising 98 mM NaCl, 4.7 mM KCl, 0.4 mM KH_2_PO_4_, 2 mM CaCl_2_, 0.2 mM MgSO_4_, 20 mM HEPES, 3 mM glucose, 21 mM lactic acid, and 0.3 mM sodium pyruvate. The pH value of this physiological buffer solution was adjusted to pH = 7.4 using 1 N NaOH solution. Sperm migration experiments were initiated by carefully layering a 100 µL liquified human semen sample above the gel barrier in the donor compartment. A total of 18 de-identified human semen samples were used for these in vitro studies. The sperm samples were left over from the Washington University Fertility and Reproductive Medicine Center and obtained from consenting, healthy adult volunteers by masturbation after 3 to 5 days of abstinence. The clinic confirmed that all samples met normal semen parameters according to the WHO manual [15], specifically sperm concentration (≥1.5 × 10^7^/mL), total motility (>40%), and progressive motility (≥32%). The sperm count and total motility of each donor sample used for this study were manually quantified by a blinded investigator using the Makler counting chamber (Sefi Medical instruments, Haifa, Israel). This manual method allows quantitative sperm assessment at very low sperm numbers where the reliability of computer-assisted semen analysis (CASA) equipment is low. After sperm migration across the gel barrier was allowed for 60 min at 37 °C, the number of sperm cells in the receiver compartment and their total motility were determined microscopically, once again using the Makler counting chamber. The in vitro contraceptive efficacy of the gel barrier was defined by the fraction of sperm recovered in the receiver compartment according to Equation (2) and the total motility of sperm that successfully moved from the donor compartment across the gel barrier into the receiver compartment.

SpR/SpF × 100%(2)

where SpR = total sperm count in receiver compartment after conclusion of the 60 min permeation experiment across a gel barrier and SpF = total sperm count in receiver compartment after conclusion of the 60 min permeation experiment across the filter insert without a gel barrier. It is noted that SpR and SpF were paired by donor due to the high inter-subject variability in sperm count and total motility. Various control experiments using the same experimental design were performed with different gel compositions, including 1% (*w*/*w*) methylcellulose gel, the VCF^®^ Gel, and the Phexxi^®^ gel. Individual sperm permeation experiments were repeated across each gel barrier using semen samples obtained from six different donors.

#### 4.2.3. Gel Performance In Vivo

##### Contraceptive Efficacy

Consistent with current U.S. Food and Drug Administration regulations guiding development of intravaginal contraceptive products [46], the rabbit model was selected for in vivo contraceptive safety and efficacy assessments of the bioresponsive CP4%/PVP4% hydrogel. All procedures were performed in compliance with the National Institutes of Health Guiding Principles for the Care and Use of Laboratory Animals at Charles River Laboratories (Horsham, PA, USA). Thirty virgin female New Zealand White rabbits (3.7–4.7 kg, Charles River Laboratories) were randomly assigned to three groups of ten animals each. In addition, six proven male breeder rabbits of the same source and strain were used as semen donors as described previously [47]. On the day of the experiment, the vaginal cavities of animals in all treatment groups were flushed with 5 mL of VFS, pH 4.2, to lower the pH value to the physiological acidic environment of humans. After the vaginal douche, female rabbits received 2 mL of the CP4%/PVP4% test formulation vaginally, using two back-to-back insertions of a gel-filled, blunted 1 mL tuberculin syringe as applicator (Becton Dickinson & Co., Franklin Lakes, NJ, USA). Other treatment groups included a “positive control” receiving 2 mL of the contraceptive VCF^®^ Gel containing 4% (*w*/*v*) nonoxynol-9 and a “sham control” where animals were only exposed to an empty syringe applicator. Artificial insemination of female rabbits in each treatment group was performed 15 min after vaginal administration of test or control formulations using 0.25 mL of pooled semen obtained from male breeder rabbits within 1 h of collection, which was diluted to 2.4–4.8 × 10^7^ sperm cells/mL (i.e., 6–12 × 10^6^ sperm cells per insemination) using Dulbecco’s Phosphate-buffered Saline, pH 7.4, supplemented with 0.3 g/L of bovine serum albumin. For each insemination, the semen was deposited within the vaginal canal, distal to the location of the sham, positive control, or test material application (approximately 5 cm within the vaginal canal). Immediately after artificial insemination, each female rabbit received 20 USP units/kg of human chorionic gonadotropin via the marginal ear vein. On gestation day (GD) 12, female rabbits were euthanized using an intravenous dose of sodium pentobarbital. The reproductive tract was dissected from the abdominal cavity to count embryos within the uterine horns and the number of corpora lutea in the ovaries.

##### Vaginal Safety

Maternal viability was observed at least twice daily starting 1–2 h after dose administration. All animals were weighed before dosing (GD 0), once on GD 6, and once on GD 12 before scheduled euthanasia. Detailed in-life dermal observations of the external genitalia were recorded before vaginal douche and twice following dose administration at 4 and 24 h, respectively. Erythema and edema were scored using the modified Draize technique [48]. At necropsy, the vagina, cervix with uterus, and ovaries with oviducts were collected for macroscopic evaluation of gross lesions by a blinded pathologist.

#### 4.2.4. Statistical Analysis

Statistically significant differences (*p* < 0.05) between experimental groups were evaluated using unpaired Student’s *t*-test and one-way or two-way analysis of variance (ANOVA) followed by Dunnett’s post hoc test or Tukey’s multiple comparisons test where appropriate (GraphPad Prism 10.4, GraphPad, San Diego, CA, USA)

## Figures and Tables

**Table 1:** Human sperm parameters measured before and after migration across various in vitro gel barriers established on Transwell™ inserts.

		Donor Compartment		Receiver Compartment	
Gel Barrier	Sperm Sample [N]	Total Motility [%] ^(a)^	Gel pH Value ^(b)^	Sperm Recovery [%] ^(c)^	Total Motility [%] ^(a)^
Methylcellulose (MC) gel	6	78.0 ± 8.6	7.2 ± 0.2	69.8 ± 6.9 *	74.5 ± 4.1
CP4%/PVP4% gel	6	71.1 ± 5.6	5.6 ± 0.1 *	3.7 ± 3.3 *	7.9 ± 13.7 *
VCF^®^ gel	6	70.4 ± 5.3	6.3 ± 0.5 *	1.3 ± 1.8 *	0.0 *
Phexxi^®^ gel	6	64.3 ± 8.5	6.7 ± 0.1 *	20.0 ± 6.9 *	28.0 ± 22.1 *

^(a)^ quantified microscopically using the Makler counting chamber; ^(b)^ measured at a gel/liquified semen ratio = 1:5 (*v*/*v*); ^(c)^ normalized to sperm recovery in the receiver compartment for the semen sample paired by donor and measured in the absence of a gel barrier (i.e., filter control)*;* * Significantly different from filter control (*p* < 0.001, one-way ANOVA and Dunnett’s multiple comparisons test).

**Table 2:** Safety of the CP4%/PVP4% gel in female New Zealand White rabbits after a single intravaginal application followed by artificial insemination.

Treatment (n = 10)	Gel Dose Volume [mL]	Erythema Scoring ^(a)^	Body Weight Gain [g] ^(b)^	Gross Pathology ^(c)^
Sham Control	0	Grade 1 (1/10)	128.7 ± 83.5	Vaginal discoloration (0/10)
CP4%/PVP4% gel	2	Grade 1 (2/10)	118.2 ± 70.2	Vaginal discoloration (1/10)
VCF^®^ Gel	2	Grade 1 (0/10)	70.1 ± 105.1	Vaginal discoloration (1/10)

^(a)^ assessed macroscopically four hours after vaginal gel administration using the modified Draize criteria for dermal erythema: Grade 1 = barely perceptible light redness; ^(b)^ body weight gain recorded during 12-day gestation period (mean ± SD); ^(c)^ macroscopic assessment after terminal euthanasia on GD 12.

**Table 3:** Maternal performance of female New Zealand White rabbits after a single intravaginal application of the CP4%/PVP4% gel followed by artificial insemination.

Treatment (n = 10)	Gel Dose Volume [mL]	Pregnancy	Number of Embryos	Corpora Lutea
Sham Control	0	Yes (9/10)	6.5 ± 4.9	11.3 ± 2.4
CP4%/PVP4% gel	2	No (0/10)	0	11.7 ± 3.5
VCF^®^ Gel	2	Yes (2/10)	1.7 ± 3.6	12.5 ± 3.9

## Data Availability

The original data presented in the study are openly available in FigShare (https://doi.org/10.6084/m9.figshare.30085429).

## Supplementary Materials

The following supporting information can be downloaded at: https://www.mdpi.com/article/10.3390/gels11110858/s1, Figure S1: Correlation between kinematic viscosity and work of shear measured using the TA–XTPlus texture analyzer fitted with the cone-cap assembly.
