# Supplementary material for: Bioresponsive Hydrogel for On-Demand Nonhormonal Contraception
Source: Gels. 2025 Oct 27;11(11):858. doi: 10.3390/gels11110858 (PMC12652176; doi:10.3390/gels11110858)
Supplement: Supplementary file 1 [file gels-11-00858-s001.zip › gels-3893316-supplementary.pdf]

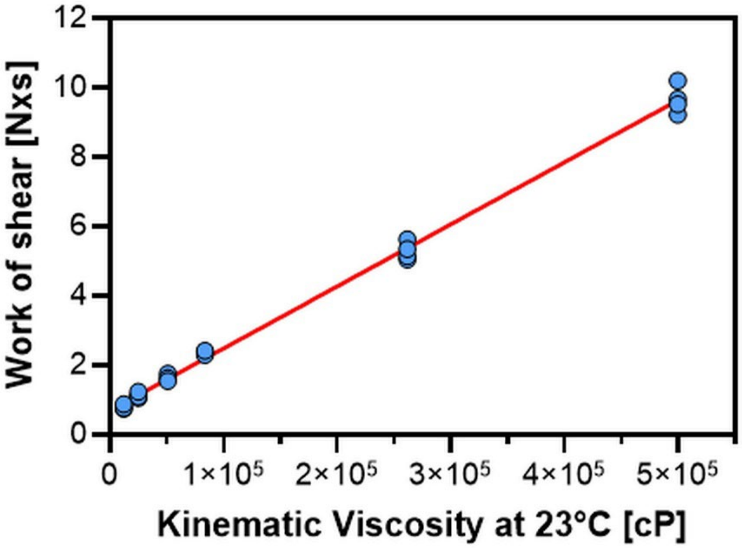

Figure S1. Correlation between kinematic viscosity and work of shear measured using the TA–XTPlus texture analyzer fitted with the cone-cap assembly. For each experiment, 0.5 mL of the viscosity standard ranging from 12,051-499,619 cP @ 23°C was added to the cup, and the work of shear was quantified under equivalent experimental conditions as described in the Methods for gel spreadability. Experiments were performed in quadruplicate. Simple statistical linear regression analysis was performed across all experimental data points resulting in the regression line shown in red ($y = 0.00001787x+0.6954$, $r^2=0.9992$).
